# Worrying about wasting GP time as a barrier to help-seeking: a community-based, qualitative study

**DOI:** 10.3399/bjgp16X685621

**Published:** 2016-05-24

**Authors:** Susanne K Cromme, Katriina L Whitaker, Kelly Winstanley, Cristina Renzi, Claire Friedemann Smith, Jane Wardle

**Affiliations:** Health Behaviour Research Centre, Epidemiology and Public Health, University College London, London.; School of Health Sciences, Faculty of Health and Medical Sciences, University of Surrey, Guildford.; Health Behaviour Research Centre, Epidemiology and Public Health, University College London, London.; Health Behaviour Research Centre, Epidemiology and Public Health, University College London, London.; Health Behaviour Research Centre, Epidemiology and Public Health, University College London, London.; Health Behaviour Research Centre, Epidemiology and Public Health, University College London, London.

**Keywords:** cancer, diagnosis, help-seeking behaviour, neoplasms, primary health care

## Abstract

**Background:**

Worrying about wasting GP time is frequently cited as a barrier to help-seeking for cancer symptoms.

**Aim:**

To explore the circumstances under which individuals feel that they are wasting GP time.

**Design and setting:**

Community-based, qualitative interview studies that took place in London, the South East and the North West of England.

**Method:**

Interviewees (*n* = 62) were recruited from a sample (*n* = 2042) of adults aged ≥50 years, who completed a ‘health survey’ that included a list of cancer ‘alarm’ symptoms. Individuals who reported symptoms at baseline that were still present at the 3-month follow-up (*n* = 271), and who had also consented to be contacted (*n* = 215), constituted the pool of people invited for interview. Analyses focused on accounts of worrying about wasting GP time.

**Results:**

Participants were worried about wasting GP time when time constraints were visible, while dismissive interactions with their GP induced a worry of unnecessary help-seeking. Many felt that symptoms that were not persistent, worsening, or life-threatening did not warrant GP attention. Additionally, patients considered it time-wasting when they perceived attention from nurses or pharmacists to be sufficient, or when appointment structures (for example, ‘one issue per visit’) were not adhered to. Close relationships with GPs eased worries about time-wasting, while some patients saw GPs as fulfilling a service financed by taxpayers.

**Conclusion:**

Worrying about wasting GP time is a complex barrier to help-seeking. GP time and resource scarcity, symptom gravity, appointment etiquette, and previous GP interactions contribute to increasing worries. Friendly GP relationships, economic reasoning, and a focus on the GP’s responsibilities as a medical professional reduce this worry.

## INTRODUCTION

International comparisons show that 1-year cancer survival rates are worse in the UK compared with countries with similar healthcare systems.^1^^,^^2^ Previous patient experience,^3^ and the perception of ‘warranting’ medical attention, can lead to delays in help-seeking.^4^ Warranting medical attention may be influenced by several factors, including the type of symptoms,^5^ awareness of the significance of the symptoms,^6^^,^^7^ perception of personal risk of cancer,^8^ and psychological, social, and physical barriers to health care.^9^

In several qualitative investigations, patients mentioned the worry of ‘wasting their GP’s time’ as a psychological barrier to help-seeking for symptoms indicative of various cancers.^10^^,^^11^ In a recent study, 15% of responders stated that worry about wasting their doctor’s time was an anticipated barrier to healthcare seeking.^12^ A qualitative synthesis showed that fear of being labelled a time-waster was a barrier to seeking help for possible cancer symptoms,^13^ which was also associated with longer anticipated help-seeking intervals.^14^ In some cases, receiving a benign diagnosis after symptomatic presentation can reinforce the fear of being a time-waster.^3^

The worry of wasting GP time reflects the discourse surrounding GP time scarcity and pressures. This perception of time-constrained doctors is enhanced by long waiting times and an apparent shortage of appointments,^15^ which may lead patients to present symptoms at a later stage, once they are confident that they are eligible for medical attention. Patients who seek help quickly have few reservations about using doctors’ time, and believe that doctors could offer reassurance,^15^ symptom alleviation, or a referral to specialist care.^13^

Informing patients of the costs of their NHS appointments or requesting a fee for visiting their GP are some of the political discussions portrayed by the media.^16^ Importantly, not understanding the underlying mechanisms that feed into the worry of wasting a GP’s time may lead to adverse effects of such media portrayal. For example, instead of increasing awareness of GP time scarcity in individuals who go to the GP unnecessarily, it may increase worry about GP time in those who are already concerned.

Although ‘wasting GP time’ is often referred to as a potent factor leading to delay in symptom presentation, this psychological barrier has not been explored in depth with people in the community experiencing cancer alarm symptoms. The aim of the present study was to examine the different meanings given to the concept of wasting GP time, by deconstructing the decision-making process that leads individuals to feel that they are wasting, or are not wasting, their GP’s time.

## METHOD

A detailed description of the study methods, including a recruitment flow chart, has been published previously;^15^ a brief summary is provided here.

**How this fits in**

Worrying about wasting GP time is a commonly cited barrier to medical help-seeking in population-based surveys, and contributes to longer time-to-symptom presentation. However, there has been very little qualitative work on the concept of wasting GP time. One qualitative study with people reporting cancer ‘alarm’ symptoms highlighted the perceived importance of limiting demands on GPs, but did not explore the concept in detail. This is the first qualitative study to explore what people experiencing cancer symptoms mean by ‘wasting their GP’s time’. The findings highlight potential avenues to mitigate against this help-seeking barrier and encourage prompt symptomatic presentation.

### Participant selection and recruitment

Participants were recruited from responders (*n* = 2042) to a large prospective postal survey conducted through four GP practices in England, which had been posted to 4913 individuals aged ≥50 years in October 2013. Responders reporting at least one cancer alarm symptom (*n* = 936/2042; 46%), and who had agreed to be contacted again (*n* = 602/936; 64%), were sent a follow-up survey after 3 months to capture symptom experiences over time; 75% of people (450/602) returned the survey. Inclusion criteria for the in-depth interviews were: experience of at least one of 14 ‘alarm’ symptoms at the 3-month follow-up (271/450; 60%), and consent to be contacted for interview (215/271; 79%). Of the 215 responders meeting the inclusion criteria, 144 were consequently invited to interview, with a response rate of 60% (86/144). Invitees were purposively sampled based on sex, age, and geographic area of residence.^3^ Interviews ceased once data saturation was achieved (*n* = 62).

### Interviews

Interview data were collected from people reporting ongoing cancer ‘alarm’ symptoms. After the initial narrative account, a semi-structured topic guide was used to ensure topics such as help-seeking and psychological barriers (wasting GP time) were explored. Prompts were used to initiate responses to the ‘wasting GP time’ component if it did not arise spontaneously. Participants were asked to tell whether they had had similar experiences to other interviewees; for example, ‘*Some people mentioned that they avoid or delay seeing the GP because they worry about wasting the doctor’s time or bothering the doctor. Does this ever apply to you?*’ Interviews lasted on average 42 minutes (range: 22–66 minutes).

### Analysis

Demographic and basic frequency information was analysed using SPSS version 21.0. Interviews were digitally recorded and transcribed verbatim. Two of the authors read and reread the transcripts and allocated arising themes to the two following questions: ‘*When do people feel as though they are wasting GP time?*’ and ‘*What are the accounts of people who use GP services freely?*’ Transcripts were coded using NVivo 9.0 software, and the emerging themes were managed in Microsoft Excel. Emerging sub-themes were discussed in frequent meetings, and agreed by the co-authors using an iterative process.

## RESULTS

The demographic characteristics of the sample can be seen in Table 1. The type of symptoms and help-seeking reported in the interview sample have been reported previously.^15^ The most common symptoms were persistent cough/hoarseness (27%) and persistent change in bladder habits (24%). The least common symptoms were unexplained bleeding and blood in urine (both 3%). Table 2 illustrates six subheadings and 11 themes that arose under the two questions, supported by participants’ quotations.

**Table 1.** Demographic characteristics of participants (*n* = 62)

**Variable**	***n* (%)**
**Sex**	
Male	33 (53.2)
Female	29 (46.8)

**Age, years**	
Mean (range)	65 (50.3–92.7)

**Ethnicity**	
White/white British	57 (91.9)
Other	5 (8.1)

**Educational level^a^**	
University degree	28 (45.2)
Higher qualification below degree	13 (21)
Lower qualification	21 (33.9)

a *Lower qualification* =*O Level/GCSE, no formal qualifications. GCSE* =*General Certificate of Secondary Education.*

**Table 2.** Experiences of wasting GP time

**Heading**	**Subheading**	**Theme**	**Example quotations**
**Situations in which people feel they are wasting their GP’s time**	When time constraints are visible	Long waiting times and difficulty making appointments	‘*If you have to wait a week for an appointment anyway they are obviously very busy, and that sort of thing would worry me, I wouldn’t want to waste a GP’s time*.’ (P13, M, 69 years; sore that does not heal) ‘*Like with most of the surgeries, it’s difficult to get an appointment with a local GP. So you think, I won’t bother them, I won’t bother them*.’ (P20*,* M, 67 years; persistent cough or hoarseness) ‘*Well, we’re used to but it’s so difficult to see your own doctor here, it’s very difficult to see your own doctor.*’ (P24, F, 64 years; persistent cough or hoarseness)
		Negative GP interactions deter, positive GP interactions encourage help-seeking	‘*I don’t know what the doctor feels. I don’t want to waste her time. But I don’t consciously waste her time.*’ (P38, F, 77 years; change in bowel habits, change in bladder habits) ‘*As I say, in the last 3 months she’s been making appointments for me to make sure I attend.*’ (P38, F, 77 years; change in bowel habits, change in bladder habits)
		One item per visit/sticking to 10 minutes	‘*I try not to barrage them with lots of things because I know it’s only a 10-minute appointment*’ (P40, F, age missing; change in bladder habits, difficulty swallowing, persistent cough, rectal bleeding) ‘*I don’t want to take up too much of their time.*’ (P53, M, 58 years; unexplained lump) ‘*I suppose you do feel time-constrained. I mean, certainly the last time I went, because I felt I could fit something in within that time, I mentioned it. So you have, like, an awareness of the amount of time that’s there and then it’s almost seeing what happens during that*.’ (P6, F, 61 years; persistent unexplained pain, unexplained lump)
	When symptoms are perceived as not serious enough	Symptom characteristics not disrupting/life-threatening/persistent	‘*But now, you see, that’s the sort of thing that I think oh I won’t bother to tell the doctor, it’s peripheral.*’ (P16, F, 77 years; change in bladder habits, abdominal bloating, change in bowel habits) ‘*It’s more that this isn’t something serious enough to bother a doctor with.*’ (P27, M, 62 years; difficulty swallowing) ‘*But then you feel, if you are not actually suffering, are you wasting the GP’s time by going down and asking if you can, you know …*’ (P14, M, 68 years; abdominal bloating) ‘*Well, persistence of the symptoms, say, sort of longer than 3 or 4 weeks. Or worsening of the symptoms. Then I would go and see a doctor. Well, it’s just to see how things settle down. I don’t want to go unnecessarily*.’ (P39, F, 64 years; change in bowel habits, blood in urine) ‘*I don’t think you should go to the doctor’s as soon as you are not feeling well, but if there’s something that’s persistent, then yeah*.’ (P50, F, 63 years; abdominal bloating, persistent unexplained pain)
		Symptoms that have previously received medical attention	‘*I mean, if you are in really dire pain or really, really worried about yourself, then you’d go back quicker than if it was something that you might think, well, perhaps giving it a bit longer, it might right itself.*’ (P37, F, 88 years; unexplained weight loss, change in bladder habits, persistent cough or hoarseness, abdominal bloating). ‘*I have the arthritis, and I have so many aches here, there, and everywhere. And the GP, there’s nothing he can do. I am already taking medications for that through the hospital, you know. So I don’t bother the GP with all my symptoms every time, just what I think is pertinent*.’ (P19, F, 67 years; unexplained weight loss, persistent cough or hoarseness)
		Comparing with hypothetical others or others with similar diagnoses	‘*I think it would be my own feeling that it is not a very big problem and lots of people that go to the doctor have far worse things, you know.*’ (P59, F, 65 years; abdominal bloating) ‘*I think GPs are generally overstretched, overworked. And I think if there are people who are more generally ill, in need of doctors, then they shouldn’t really tie up resources.*’ (P7, M, 51 years; persistent cough or hoarseness, rectal bleeding) ‘*And quite often Saturday appointments you, sort of, avoid because you think that’s really for emergency or something very serious.*’ (P7, M, 51 years; persistent cough or hoarseness, rectal bleeding) ‘*So then you back up again and you think, okay, I won’t take the emergency appointment because there’s somebody who might really need that. So you wait. And that’s not good either.*’ (P21, F, 67 years; change in bowel habits, abdominal bloating, change in appearance of mole)
	When an alternative healthcare practitioner could provide necessary diagnosis or treatment	Nurse practitioner or pharmacist can offer suitable support	‘*When you walk in* [to see the nurse]*, you feel at ease, whereas when you walk in to the doctor’s you feel, “Well, I’ve got to be going in about 3 minutes because I’m wasting his time.” And I mean, I probably spend exactly the same time with her, but I don’t have that pressure, or I don’t feel that I have that pressure*.’ (P24, F, 64 years; persistent cough or hoarseness) ‘*So my first thought would be to try to deal with it myself by using a patent medicine, or whatever, and, if that doesn’t work, then go to the GP.*’ (P5, M, 62 years; sore that does not heal)

**Accounts and beliefs of people who do not feel that they are wasting their GP’s time**	It is the GP’s responsibility to be available to those in need	GP responsibilities	‘*A doctor is not there just for Christmas … What on earth is a doctor there for other than to look after patients*?’ (P11, M, 69 years; change in bowel habits) ‘*I think that would have been true for me more so many years ago, but I think less so now because, after all, the doctor is there to do their job. So they are doing a job and you need to look on it that way. But I know if I go right back in history, obviously coming from Scotland we had this very old-fashioned view that you didn’t trouble the doctor unless it was serious. So somewhere in my psyche, that’s probably still there.*’ (P21, F, 67 years; change in bowel habits, abdominal bloating, change in appearance of mole)
	GPs offer a service financed through taxes paid by patients	Financial contributions towards medical services	‘*I mean, they are paid to do a service and that’s how it is.*’ (P5, M, 62 years; sore that does not heal) ‘*I don’t feel overawed or deep reverence of my doctor. I mean, they are paid to do a service and that’s how it is.*’ (P5, M, 62 years; sore that does not heal) ‘*They are well paid, doctors.* [They] *are a very well paid profession since Mr Blair looked after the profession*.’ (P11, M, 69 years; change in bowel habits)
		Awareness of adequate service use	‘*I wouldn’t like to abuse the service*.’ (P11, M, 69 years; change in bowel habits) ‘*I don’t want to be a time-waster. But having said that, I’m very aware that I’ve paid a great deal of money into the service and I’ve had very little back.*’ (P44, M, 54 years; change in appearance of mole, unexplained lump)
	GPs care about their patients	Positive relationship with GP	‘*No, no. I mean, he encourages it, you know, “If there’s any problems, come and see me.” So I don’t have any problems that way at all. And I’m not certainly an NHS basher, or anything like that. It’s just that that’s the way it is.*’ (P2, M, 74 years; change in bladder habits)

### When do people feel like they are wasting GP time?

#### When time constraints are visible

When discussing their reservations about seeking help for cancer ‘alarm’ symptoms, participants made frequent reference to the time constraints incurred by GPs. Reference was made to increasing duties on GPs alongside their medical responsibilities such as ... ‘*all the … paperwork they have got to do and the money juggling that the government is forcing on them’.* (P18, M, 50 years; change in bowel habits, rectal bleeding)

Participants were understanding of GPs and how ‘... *they are not going to have time to do this, that, and the other. It’s the system.*’ (P29, F, 83 years; changes in the appearance of a mole), and mentioned how this made them ‘... *reluctant to be knocking on the GP’s door all the time because we know how busy they are.*’ (P21, F, 67 years; change in bowel habits, abdominal bloating, change in the appearance of a mole).

The perception of GPs’ lack of time was derived in particular from waiting times at the practice and difficulty making an appointment: ‘*They have got a hard enough job as it is. As I say, you’ve got to wait a week for an appointment anyway.*’ (P22, M, 68 years; persistent cough or hoarseness)

Furthermore, negative interactions where participants were left with the impression that they were ‘*crying wolf too much*’ (P11, M, 69 years; change in bowel habits) made participants feel as though they were wasting their GP’s time. At times, this made help-seeking humiliating and made some feel as though GPs ‘*are just not interested*’ (P54, F, age missing; persistent unexplained pain): ‘*Well, it was just so overwhelmingly humiliating, the fact that I went in and said, “Please can you do something with this?” And he literally turned round and said, “Why are you bothering me with something as trivial as that?”*’ (P24, F, 64 years; persistent cough or hoarseness)

Although negative experiences with GPs led some to feel bad about help-seeking, others reported how positive GP interactions reduced their worries about wasting time, pointing out that the GP ‘*doesn’t make you feel that you’re a nuisance.*’ (P20, M, 67 years; persistent cough or hoarseness)

Some conveyed a need to avoid being perceived as a hypochondriac when returning with a symptom. They expressed how they did not want to bother the doctor: ‘*I do feel as though I am … not annoying them, but “oh not you again”, sort of thing.*’ (P62, M, 62 years; persistent unexplained pain, abdominal bloating)

In some cases, such contemplation occurred despite acknowledging that they did not know how the doctor felt or claiming that they were ‘*not scared of what he* [GP] *might think of me, exactly, but I don’t want to bother him.*’ (P36, F, 72 years; sore that does not heal, persistent unexplained pain)

Participants mentioned that: ‘*It’s quite important to respect the fact they have these very limited appointments now. So I try not to take advantage of that and mention anything else.*’ (P42, F, age missing; change in bowel habits, persistent cough or hoarseness)

Thus, to avoid wasting GP time, participants not only held back information so that they would not compromise their GP’s appointment structure, but some also felt they had to be apologetic if they did not stick to the ‘one item per visit’ rule or the 10-minute time slot. One participant said he would: ‘*rehearse what you are going to tell the doctor, because I’m very, very conscious of they’ve only got so much time for each patient.*’ (P53, M, 58 years; unexplained lump)

#### When symptoms are perceived as not serious enough

Some participants felt that seeking help for symptoms that were not serious enough would result in wasting GP time. Reference was made to symptom characteristics, such as whether they were life-threatening, treatable, or chronic. Some said they ‘... *wouldn’t want to go and see them* [GP] *with something which was trivial*’ (P5, M, 62 years; sore that does not heal) or ‘*It’s got to be life-threatening, and that requires investigations.*’ (P22, M, 68 years; persistent cough or hoarseness)

Some described withholding information about less serious symptoms, believing they would otherwise waste their GP’s time. For example, they made reference to not wanting to: ‘... *bother the GP with all my symptoms every time, just what I think is pertinent’.* (P19, F, 67 years; unexplained weight loss, persistent cough or hoarseness)

Where long waiting times existed, there appeared to be a link between awareness of GP time constraints, symptom seriousness, and the worry of wasting GP time, and potentially benign symptoms increased worry over wasting GP time. Some participants felt that: ‘... *the longer the waiting list, the more urgent I think it needs to be in order to go.*’ (P9, M, 63 years; change in bladder habits, sore that does not heal)

Many participants only felt comfortable seeking help for symptoms that were ‘worsening’ or ‘persistent’, as these characteristics were seen to reduce the likelihood of going unnecessarily. Although ‘persistent’, symptoms could also be problematic because people do not want to go back unless ‘*you are in really dire pain.*’ (P37, F, 88 years; unexplained weight loss, change in bladder habits, persistent cough or hoarseness, abdominal bloating)

Symptom characteristics also contributed to how individuals compared their symptoms or situation to hypothetical others who may be in more urgent need of medical attention. Taking hypothetical others into account increased their worry of wasting their GP’s time. Some felt uncomfortable ‘... *bothering the doctor, they have got people who are much more seriously ill to see to than I.*’ (P10, M, 77 years; abdominal bloating, persistent cough or hoarseness), and that ‘... *other people have more serious things wrong. My things are just trivial.*’ (P17, M, 62 years, change in bladder habits)

#### When an alternative healthcare practitioner could provide a diagnosis or treatment

Participants felt that sometimes an alternative healthcare practitioner, such as a nurse, a pharmacist, or even self-medication, could provide a diagnosis or treatment, and that GP time was wasted were these alternative sources of medical attention not sought before a visit to the GP. Going to the pharmacy or self-medicating was a way for participants to ‘*cope with it elsewhere*’ (P27, M, 62 years; difficulty swallowing) and to ‘*take care of this myself.*’ (P19, F, 67 years; unexplained weight loss, persistent cough or hoarseness)

Nurse practitioners were seen as ‘*quite useful for going along with something that you just want reassurance about.*’ (P40, F, age missing; change in bladder habits, difficulty swallowing, persistent cough or hoarseness, rectal bleeding)

Reference was made to how participants felt pressured during GP appointments compared with feeling at ease with a nurse. Participants would categorise their symptoms and reasons for a healthcare visit. If symptoms were not serious, and if the outcome sought was reassurance, then visiting a nurse or pharmacist was considered appropriate.

### What are the accounts from participants who use GP services freely?

#### It is the GP’s responsibility to be available to those in need

Those who used GP services freely focused on the GP’s responsibilities and obligations as a healthcare provider, but also as an employee of the government through the NHS. These individuals felt that the GP’s responsibility is primarily to: ‘... *help us when we* [are in] *need. So it’s not a question of bothering the doctor.*’ (P19, F, 67 years; unexplained weight loss, persistent cough or hoarseness)

Some participants argued that ill people secured the GP’s job and that seeking help is therefore ‘... *not wasting his time; he is there to do this job. After all, if it was not for us sick people there, what would the GP do*?’ (P2, M, 74 years; change in bladder habits)

This rationalisation of the GP’s role strengthened their attitude: ‘*If I want to go, I go.*’ (P38, F, 77 years; change in bowel habits, change in bladder habits)

#### GPs offer a service financed through taxes paid by patients

A further justification for using GP services freely was the observation that GPs offer a service financed by taxpayers. Participants felt ‘*entitled to go to the GP*’ because ‘*I pay my taxes. I’ve got a pension and I pay a hefty 20% of that.*’ (P58, M, 74 years; change in bladder habits, change in appearance of mole), and because ‘*I’m wanting some sort of reassurance and I’m paying for it.*’ (P30, F, 92 years; persistent cough or hoarseness, change in appearance of mole)

The worry of wasting GP time became secondary to those who felt that medical attention was justified through them having paid towards the NHS in taxes or towards private insurance.

#### GPs care about their patients

Those who used GP services freely reported having a friendship-like relationship with their GP: ‘*I go because I made friends with my doctor.*’ (P3, M, age missing; persistent unexplained pain) They also described confidence in their GP. Those who sought help freely believed that GPs care about their patients: ‘*He encourages it* (help-seeking)*, you know, “If there’s any problems, come and see me”*’ (P2, M, 74 years; change in bladder habits)

Some reported that their GP made appointments for them to ensure that they returned regularly: ‘*And she* [GP] *makes the appointment, I don’t make the appointment.*’ (P38, F, 77 years; change in bowel habits, change in bladder habits)

## DISCUSSION

### Summary

As far as the authors are aware, this is the first study to carry out an in-depth investigation about what people experiencing persistent cancer alarm symptoms mean by ‘wasting their GP’s time’ and explore situations in which participants felt that they were wasting their GP’s time. Understanding how the worry of wasting GP time influences the appraisal process and help-seeking decisions is crucial to improving early symptomatic presentation.

Symptom characteristics strongly influenced whether participants felt they would waste their GP’s time or not. However, awareness of time and resource constraints by the GP and the NHS also influenced patients’ perception of wasting their GP’s time. An awareness of long waiting times and difficulty making appointments led participants to report an increased worry over wasting their GP’s time. Participants felt that the longer the waiting times for appointments, the more serious the symptom(s) needed to be. This reasoning offers novel insight into how various components that contribute to the worry of wasting GP time interact with each other. Resource constraints also led to a heightened awareness of what the GP may think of patients for seeking help for particular symptoms. Some participants had concrete ideas of what the GP may think or feel about them despite acknowledging that they did not actually know.

Importantly, this study also investigated the accounts of those who use GP services freely. These individuals felt that due to the taxes they had paid, they were entitled to use the service with moderation and had a general belief that the GP’s responsibility was to help those in need and that GPs generally cared about their patients. They also recounted how a good relationship with their GP reduced their worry of wasting their GP’s time.

### Strengths and limitations

This study builds on a growing body of research investigating influences on the patient interval (time from noticing a bodily change to seeking medical help),^17^^,^^18^ to support individuals to seek medical advice as soon as possible — a key element of the UK government’s strategy for cancer,^19^ and the Independent Cancer Taskforce report.^20^ This study offers novel and more detailed insights into the complexities underlying ‘wasting GP time’ as a barrier to help-seeking. By interviewing people with ongoing cancer symptoms, but without mentioning cancer, the possible bias introduced in retrospective studies with cancer patients was reduced.^11^ The prospective nature of the present study also meant participants had experienced symptoms for at least 3 months before they were interviewed, reducing the possibility that symptoms were short-lived or transient.

The sample was drawn from London, and South East and North West England, which limits generalisability to other regions. It was also beyond the study’s scope to examine the effect of specific symptoms on patients’ worry of wasting their GP’s time. Investigating patients with more homogeneous symptom groups represents an area for future research; it could provide a more detailed picture and offer a powerful focus for intervention. Another outstanding question is how to balance minimising worry about wasting GP time in those who perceive it as a barrier, rather than readily encouraging all people to contact their GP more often for advice. The study also focused on older men and women (aged ≥50 years) because of their higher risk of developing cancer.^21^ However, certain demographic subgroups may be more likely to report worry about wasting their doctor’s time, which could be a consideration for future research. For example, in a Danish study, a younger age group (30–49-year-olds) were more likely to report worry about wasting their doctor’s time than older age groups.^12^ Finally, GPs were not interviewed and patient–GP interactions were not observed; thus, the GP perspective is notably absent in this account. Future research could interview patient–GP dyads to further explore how interactions may influence help-seeking.

### Comparison with existing literature

Wasting GP time is a barrier to help-seeking frequently mentioned by individuals experiencing cancer ‘alarm’ symptoms.^10^^,^^11^^,^^14^ As observed in the existing research, participants in the present study felt that, until the symptoms display a certain gravity or until they could categorise them as life-threatening, help-seeking would be seen by themselves and others as wasting their GP’s time.^11^ Participants sought to protect their self-identity from being labelled a hypochondriac or ‘time-waster’ by avoiding help-seeking for symptoms that could be benign, and for symptoms following an ‘all-clear’ diagnosis,^3^ using health care only when they felt it was absolutely necessary. These findings support previous research. 3^,^^13^^,^^22^

Previous research also showed how physician interaction can influence help-seeking behaviour, by legitimising help-seeking.^23^ In the present study, where GPs came across as abrasive and uninterested, worrying about wasting GP time increased, whereas friendly encounters reduced concern over wasting GP time. Positive interactions with GPs were described in the present qualitative study, in contrast to previous research where GPs were only mentioned as barriers to help-seeking.^11^

A novel finding was related to the worry of taking GP time away from hypothetical others who may be in more acute need of medical attention. This was a dominant theme during the interviews and highlighted how, when participants compared their own symptoms with those of hypothetical others who were ‘much more seriously ill’, the worry of wasting GP time increased.

In line with previous research, participants felt they should seek advice from alternative healthcare providers before consulting their GP,^11^ although in the present study this was in the context of not wanting to waste their GP’s time, rather than lack of confidence in their GP.

### Implications for research and practice

The appraisal processes of what behaviour constitutes wasting GP time is made up of sociocultural and experience-driven factors that vary from person to person. For practising clinicians, this study highlights areas where they might encourage appropriate help-seeking (for example, providing a safeguard for people reporting vague, non-specific symptoms by making follow-up appointments on behalf of patients). Future studies that capture the discourse of seeking help with GPs and alternative healthcare providers, such as nurse practitioners, in a more in-depth way could offer valuable insight into this promising target for intervention. Focusing on nurse practitioners as a point of contact for symptoms that may not fulfil the subjective requirements for GP attention may not only reduce the barrier represented by ‘worrying about wasting GP time’, but may also help to distribute demands on GPs’ time and resources.

An interesting area for future research would be to find out more about cognitive and affective constructs surrounding ‘hypothetical others’, and what part the media play in informing these constructs.

This study on adults reporting cancer ‘alarm’ symptoms highlighted that the worry over wasting GP time was influenced by cognitive aspects (symptom characteristics and financial contributions to the NHS, GP responsibilities, in-appointment conduct) and emotive aspects (needs of hypothetical others, relationship with GP). Research is needed on how these attitudinal and emotive factors could be addressed in public health campaigns aimed at reducing time to presentation and improving earlier diagnosis of cancer.
